# Meta‐Analysis of Iron Excess Stress in Rice: Genes and Mechanisms of Tolerance to Acidic Soil

**DOI:** 10.1111/ppl.70473

**Published:** 2025-08-27

**Authors:** Divya Gupta, Sanjib Kumar Panda, Petra Bauer

**Affiliations:** ^1^ Plant Functional Genomics and Molecular Biology Laboratory, Department of Biochemistry Central University of Rajasthan Ajmer Rajasthan India; ^2^ Institute of Botany Heinrich Heine University Düsseldorf Germany; ^3^ Cluster of Excellence on Plant Science (CEPLAS) Heinrich‐Heine‐University Düsseldorf Germany

**Keywords:** acidic soil, antioxidants, iron, meta‐analysis, rice, toxicity

## Abstract

Iron toxicity, predominantly stemming from excessive levels of ferrous iron (Fe^2+^) in acidic soils, poses a considerable challenge for crop production. Hypoxic conditions induced by waterlogging can exacerbate Fe^2+^ availability, which significantly impacts the cultivation and productivity of rice ( *Oryza sativa* ), a staple food for millions worldwide. In several regions across South America, Africa, and Asia, the prevalence of acidic soils results in elevated Fe^2+^ levels leading to iron toxicity, thereby hindering rice yield. Some regional rice varieties demonstrate a notable adaptation to high iron conditions, offering insights into the tolerance mechanisms through comparative physiology and transcriptomic studies. This review synthesizes the various strategies employed by rice plants to mitigate iron toxicity stress, with a focus on the regulation of essential genes and genetic pathways associated with iron transport and homeostasis. We place particular emphasis on the co‐expression networks and predicted subcellular localization of the proteins encoded by these genes. A meta‐analysis of differential gene expression data gathered from studies involving six distinct rice lines—either tolerant or sensitive—reveals significant influences of plant genotype, developmental stage, and treatment type on the expression patterns, leading to the identification of robust marker genes associated with the iron excess response. Our comprehensive literature review uncovers several critical knowledge gaps, establishing a framework for developing novel approaches aimed at elucidating the molecular mechanisms underpinning iron stress tolerance. These insights are vital for enhancing rice yield in iron‐rich, acidic soils, ultimately contributing to improved food security in affected regions.

## 1 Introduction

**FIGURE 1:**
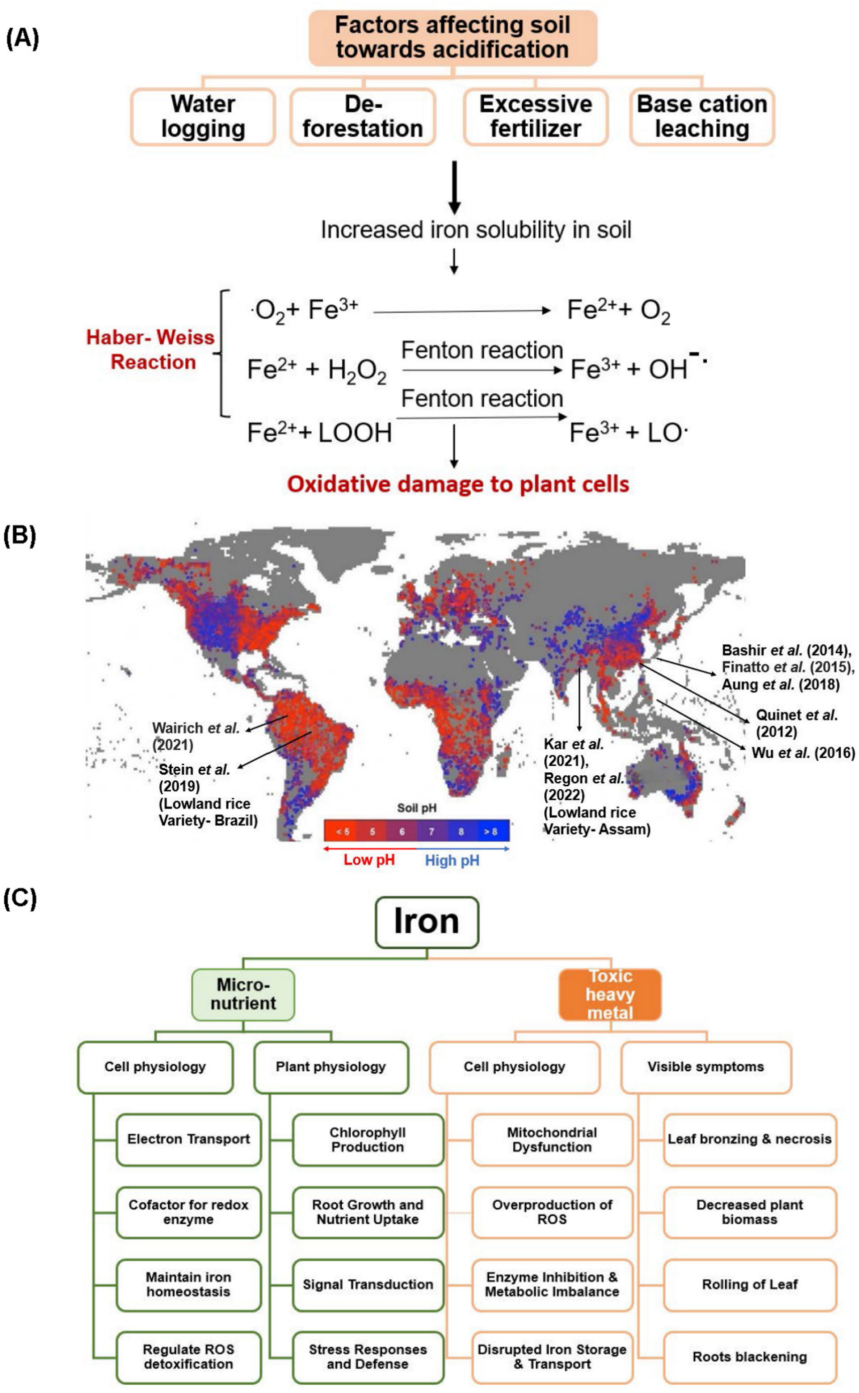
Overview of iron (Fe) toxicity stress in rice. (A) Top, Factors affecting soil pH and Fe toxicity, caused by, bottom, Haber‐Weiss, and Fenton reactions. (B) World map representing soil pH conditions worldwide, with indicated origins of different studied rice varieties exposed to Fe excess and respective references. (C) Effects of Fe as micronutrient and toxic heavy metal on plant growth and development.

Various soil properties and environmental factors significantly influence iron (Fe) availability to plant roots, with soil pH playing a key role (Figure 1A; Box 1). Well‐aerated soils maintain iron in its ferric form (Fe^3+^), promoting iron homeostasis under optimal conditions (Wairich, Aung, et al. 2024). However, at low pH, iron shifts to its more soluble ferrous form (Fe^2+^), which can become toxic and cause irreversible damage to the growth of non‐adapted plants (Becker and Asch 2005; Aung et al. 2018; Wairich, Aung, et al. 2024), a condition encountered primarily in regions of Southeast Asia, Sub‐Saharan Africa, and South America (Figure 1B).

In these regions, iron toxicity in rice ( *Oryza sativa* ) is a significant ecological and agricultural challenge that affects millions of smallholder farmers. This staple food for over half the global population is predominantly cultivated in regions where waterlogged conditions are common—either due to environmental factors or as a deliberate cultivation choice—and its consumption has steadily increased over the years (Figure 1B). As a semi‐aquatic plant, rice is predominantly cultivated in flooded or waterlogged conditions, making it highly susceptible to soil and water‐related constraints, including nutrient imbalances and metal toxicities. Unlike deep‐rooting upland rice varieties grown in dry fields, lowland rice varieties are generally grown in a paddy system, thus more exposed to Fe excess. The threshold for Fe toxicity in rice varies widely, with concentrations ranging from 10 to over 2000 mg L^−1^ in the soil solution, which is roughly 4 to 700 times more Fe than is present in a standard hydroponic growth medium. This variation is influenced by soil geochemistry, nutrient dynamics, and rice varietal differences (Becker and Asch 2005). With climate change altering rainfall patterns, the frequency of waterlogging and associated iron toxicity is expected to rise, emphasizing the urgent need for stress‐tolerant rice varieties. Understanding the natural mechanisms of iron stress responses in rice varieties that are tolerant to iron excess is crucial for developing effective mitigation strategies.

### 1.1 Properties of Acidic Soils

Excess Fe is commonly associated with waterlogged soils but can also occur across various soil types, including Ferralsols (Fe‐rich, acidic tropical soils), Gleysols (Waterlogged Soils), Acrisols (Red and Lateritic Soils), Podzols (Leached Soils), and Fluvisols (Alluvial Soils) (Vose 1982), followed by some other factors, such as deforestation and excessive use of fertilizers (Figure 1A). Deforestation accelerates soil acidification, waterlogging, and nutrient imbalances, all of which contribute to increased iron toxicity in agricultural lands, while excessive fertilizer application can indirectly increase iron toxicity risk by acidifying soil and altering nutrient balance. Globally, the distribution of acidic soils varies across regions, with America having the highest proportion at 40.9%, followed by Asia at 26.4%, Africa at 16.7%, Europe at 9.9%, and Australia and New Zealand at 6.1% (Figure 1B) of the total 4.5% of the acidic soil that is distributed in arable lands (Ngoune Tandzi et al. 2018). Acidic soils in India are predominantly found in the humid southwestern, northeastern, and Himalayan regions. In the Northeast, approximately 95% of soils are acidic, with nearly 65% having a pH below 5.5, indicating strong acidity (Mishra et al. 2020). In tropical South America, 85% of the soils are acidic. Across all soils, only 0.1% have a pH of 7.3, while 75% of the subsoil has a pH of 5.3 (Fageria and Nascente 2014). In Africa, Acrisols and Ferralsols collectively cover a substantial portion of the continent's land area, accounting for 2.9**%** and 10.3% of the total surface, respectively. Soil acidity is increasingly becoming a widespread issue, both in terms of geographic expansion and severity, particularly in sub‐Saharan regions. For instance, in Ethiopia, approximately 43% of cultivated land is affected by soil acidity. Of this, nearly 28% is classified as strongly acidic, with a pH ranging between 4.1 and 5.5, posing significant challenges to agricultural productivity and soil fertility (Agegnehu et al. 2021).

### 1.2 Numbers on Rice Cultivation

In the 2023/24 crop year, worldwide rice consumption reached approximately 523.8 million metric tons, marking a rise from 437.18 million metric tons in the 2008/09 crop year (Statista 2024–25). According to the U.S. Department of Agriculture (USDA)‐Foreign Agriculture Service (2024–25), India is the world's second‐largest rice producer, accounting for 27.2% of global production, following China, the leading producer. Brazil ranks 10th, contributing 1.5%, while Nigeria holds the 14th position, producing 1% of the world's rice supply.

## 2 Fe Toxicity Effects and Symptoms

Fe is a crucial micronutrient involved in numerous biochemical and physiological processes essential for plant growth and development. The importance of Fe lies in its ability as a redox catalyst, and as such, it is used as a cofactor in the form of heme‐Fe, Fe‐sulfur clusters, or free Fe oxide for enzymatic redox and electron transfer reactions. Thereby, it plays a vital role in basic cellular processes such as metabolism, for example nucleotide metabolism, and chlorophyll biosynthesis. Fe is integral to energy production through respiration and photosynthesis, facilitating the electron transfer in the mitochondrial and chloroplast electron transport chains (Rout and Sahoo 2015). Chloroplasts are typically rich in iron (Fe), and ferritin‐Fe complexes—structures where iron is safely stored within the ferritin protein—are also found within the chloroplasts themselves (López‐Millán et al. 2016). The reactive properties of Fe render it toxic at high concentrations since free Fe catalyzes non‐enzymatically the generation of hydroxyl and lipid alkoxyl radicals through the Haber–Weiss and Fenton reactions (Le et al. 2019; Figure 1A). These radicals are the most potent reactive oxygen species (ROS) and cause severe oxidative stress, membrane damage, macromolecule deterioration, and ion leakage (Hossain et al. 2012). Most importantly, plants do not have a specific detoxification for these radicals, and the regular antioxidant system is insufficient. Iron toxicity in rice manifests through a range of symptoms, primarily affecting the leaves, roots, and overall plant growth (Figure 1C). Initially, tiny brown spots appear on the lower leaves, starting from the tips and gradually spreading toward the leaf base. As the condition worsens, these spots merge along the intervein, turning the leaves orange‐yellow to brown and eventually leading to leaf death. This symptom is known as “leaf bronzing”. In some varieties, the leaf tips dry up after turning orange‐yellow, while in severe cases, leaves may appear purple‐brown. Leaves also roll and bend towards the inside, which resembles a drought symptom (Kar et al. 2021). Affected plants exhibit stunted growth, poor tillering, and narrow leaves, which often remain green. Root systems are coarse, sparse, and damaged, with a dark brown to black coating and many dead roots, termed “root blackening” (Dobermann 2000).

Fe toxicity rarely occurs in isolation and is often compounded by other abiotic stresses, including phosphorus deficiency, salinity, and drought. Alternating dry and flooded conditions destabilize the redox balance in the soil, causing fluctuations in Fe forms and amplifying stress (Becker and Asch 2005; Aung and Masuda 2020). Excess Fe also disrupts the uptake of essential nutrients like phosphorus, potassium, and zinc, further impairing plant growth. In saline environments, waterlogging exacerbates ionic toxicity, worsening oxidative damage. A widespread issue in acidic soils is aluminum (Al) toxicity, resulting from the increased solubility of Al^3+^ ions under low pH conditions (Bojórquez‐Quintal et al. 2017). In many affected regions, such as South Asia and Sub‐Saharan Africa, Al toxicity frequently co‐occurs with iron (Fe) toxicity, compounding stress on rice plants (Kar et al. 2021). While both stresses lead to root growth inhibition and oxidative damage, they operate through distinct physiological mechanisms: Fe toxicity is primarily linked to redox cycling and the generation of reactive oxygen species (ROS), whereas Al toxicity disrupts cell wall structure, interferes with nutrient uptake, and impairs root tip function (Kar et al. 2021; Rahman et al. 2024). Despite their differences, both stress responses share common elements such as activation of antioxidant defenses and altered metal transporter activity. Understanding these converging and diverging mechanisms is critical for developing rice varieties with enhanced tolerance to multiple soil constraints in acidic environments.

## 3 Rice Genetic Variation in Acidic Soils

Genetic variation studies are powerful tools to uncover the genetically encoded adaptive mechanisms in plants. Such genetic variation studies have been conducted with rice varieties from different areas to reveal genetic mechanisms for explaining sensitivity or adaptation to acidic soil cultivation (Table 1). Certain rice germplasms have been used in physiological and transcriptomic experiments to uncover molecular responses to excess Fe (Kar et al. 2021). This includes the Indian lowland rice varieties Hacha (sensitive) and Lachit (tolerant). These two varieties were selected from the extremities of a test panel of 16 varieties with differing abilities to thrive in excess Fe in an experimental hydroponic environment (Kar et al. 2021). Aromatic rice is a popular short grain lowland rice variety generally cultivated in the northeastern region of India. The aromatic rice variety Keteki Joha is sensitive to Fe excess, and the motivation to study it was with the ultimate goal of genetic improvement through methods such as gene editing (Regon et al. 2022). Two studied Brazilian lowland rice varieties are EPAGRI 108 (tolerant) and BR‐IRGA 409 (susceptible; Stein et al. 2019). IR29 (susceptible) and FL483 (tolerant) rice varieties from the International Rice Research Institute (IRRI), Los Baños, Philippines, were studied under Fe (Wu et al. 2017). Additionally, the Japanese variety Nipponbare was examined at different growth stages under varying Fe concentrations (Bashir et al. 2014; Finatto et al. 2015). The studies on Fe stress in rice were conducted under varying pH conditions (4.0–5.5), different types and concentrations of Fe sources (FeSO_4_, FeCl_2_, Fe‐EDTA), and differing stress durations (Table 1). Thus, the experimental setups differed in multiple aspects, indicating that presumably these experimental variations, in addition to the different used germplasms, also uncovered a variety of responses. This diversity may render it difficult to recognise basic Fe tolerance mechanisms.

**TABLE 1:** Overview of comparative transcriptome studies addressing iron excess conditions in rice (*Oryza spec.*).

Age^a^	Rice variety^+^	pH	Iron salt	Fe excess concentration	Duration of stress	Tissue analyzed	Access^c^	References
24 days	I Kong Pao (IKP)	4.5	FeSO_4_	0.45 mM	3 days, 3 weeks	Root, shoot	No	Quinet et al. 2012
35 days	*O. meridionalis*	5.5	FeSO_4_	0.90 mM	7 days	Shoot	No	Wairich et al. 2021
20 days	EPAGRI 108 (tolerant) BR‐IRGA 409 (susceptible)	^b^	FeSO_4_	3.3 mM	3 days	Root	No	Stein et al. 2019
8 days	Lachit (tolerant) Hacha (susceptible)	5.2	FeSO_4_	15 mM	2 days	Root, shoot	Yes	Kar et al. 2021
17 days	IR29 (susceptible) FL483 (tolerant)	^b^	FeSO_4_	3.6 mM	4 days	Root, shoot	Yes	Wu et al. 2017
24 days	Tsukinohikari	4.0	FeCl_2_	0.36 mM to 5.36 mM	14 days	Root, shoot	No	Aung et al. 2018
14 days	Nipponbare	5.5	Fe^+2^	7 mM	18 days	Shoot	Yes	Finatto et al. 2015
3 weeks and 60–70 days	Keteki Joha	4.5	Fe‐EDTA	2.5 mM	3 days, 2 weeks	Root, shoot	Yes	Regon et al. 2022
3 weeks	Nipponbare	5.5	Fe‐EDTA	0.5 mM	1 weeks	Root, shoot	No	Bashir et al. 2014

^a^ Stress imposition age; *O. sativa* if not otherwise specified.

^b^ pH is not defined for the respective data set.

^c^ Differentially regulated gene set provided for access.

## 4 Genetic Basis for Tolerance to Fe Toxicity

**FIGURE 2:**
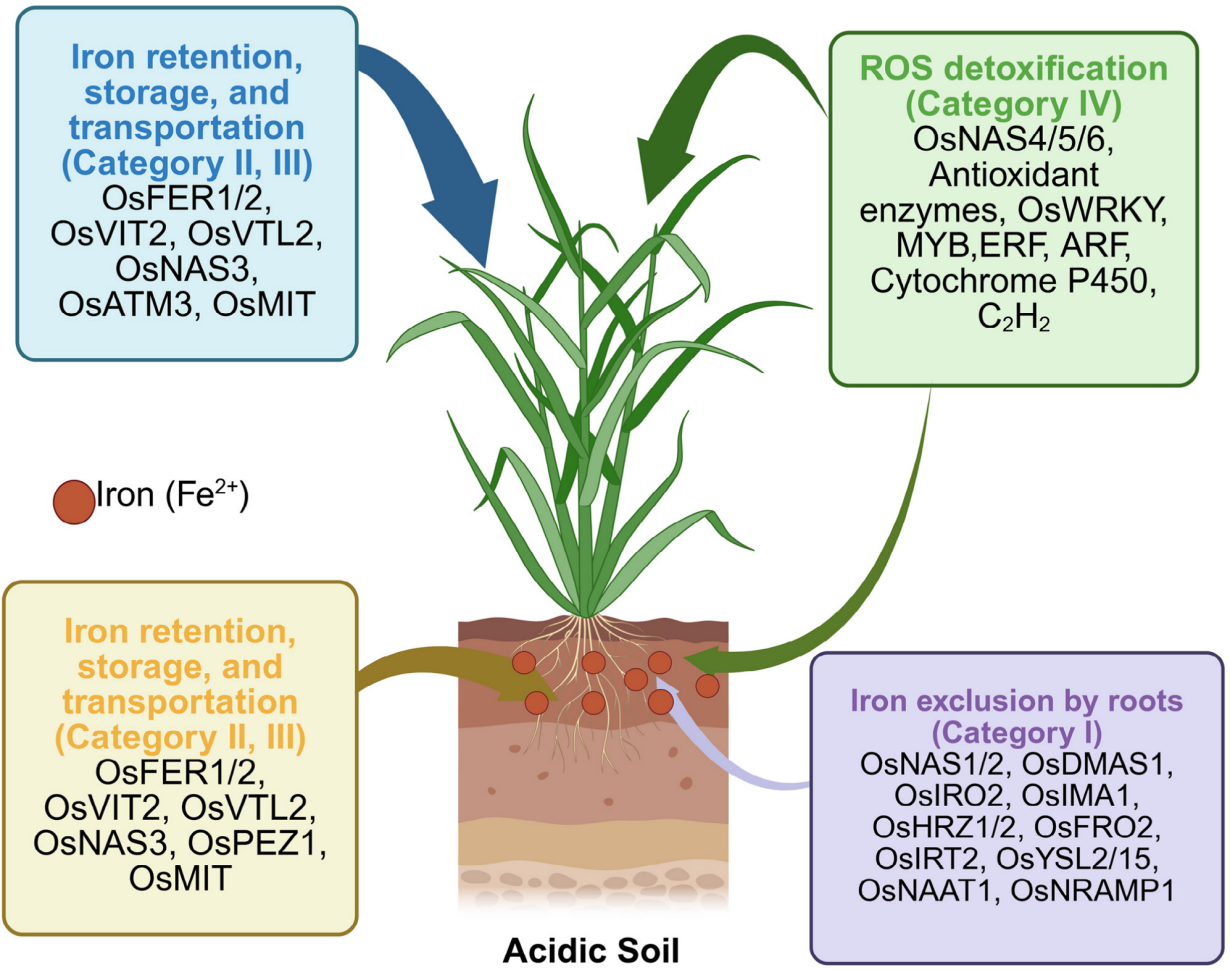
Four strategies for tolerance to iron (Fe) toxicity in rice grown in acidic soils. These four distinct strategies have been proposed by Aung and Masuda (2020). The indicated proteins are regulated at transcriptional level, and their functions depicted in Table S1. Created with Biorender.

It is crucial to understand the nature of Fe excess tolerance for breeding new resilient rice varieties. The defense strategies of rice towards delimiting Fe toxicity have been recently reviewed (Aung and Masuda 2020), and it was suggested that four distinct mechanisms may primarily account for tolerance, distinguished as four categories of responses (Figure 2, Aung and Masuda 2020). Categories 1, 2, and 3 work under mild to moderate iron excess conditions, as they mediate Fe exclusion by roots, Fe retention and sequestration in roots to prevent long‐distance transport towards shoots, as well as sequestration in leaves. Category 4, instead, works to mitigate the consequences of severe Fe stress and is ROS detoxification (Figure 2). A list of encoded protein functions is provided in Table S1.

### 4.1 Four Strategies for Tolerance to Fe Toxicity in Rice

Category 1‐Iron exclusion from roots: This strategy consists in avoiding uptake of Fe from the soil by repressing and down‐regulating Fe mobilization and acquisition (Figure 2, Table S1). In rice, this is achieved by down‐regulating the import of Fe^2+^ through divalent metal ion importers and down‐regulating the import of Fe^3+^ through phytosiderophores with the involvement of transporters for phytosiderophore export and import and enzymes for phytosiderophore biosynthesis.

Categories 2 and 3 ‐Fe retention in roots and Fe compartmentalization in shoots: These strategies serve to sequester Fe inside cells in compartments that keep Fe in a less reactive state (Figure 2, Table S1). It may include vacuolar sequestration through upregulating the vacuolar Fe import and downregulating vacuolar export (Morrissey and Guerinot 2009; Zhang et al. 2012), chelation by nicotianamine by upregulating the biosynthesis of this chelator (Inoue et al. 2003; Aung et al. 2018), and the Fe‐ferritin complexation in plastids through upregulating ferritin. Mitochondrial Fe and Fe‐S cluster homeostasis is also adjusted with the participation of mitochondrial proteins (Bencze et al. 2006; Kushnir et al. 2001; Chen et al. 2007; Bashir et al. 2011). Sequestration in roots prevents the entry of Fe into the xylem stream and its long‐distance transport towards leaves.

Category 4‐Reactive oxygen species (ROS) detoxification: This last strategy is limiting the effects of ROS produced upon iron toxicity (Figure 2, Table S1). This is achieved through rebalancing the redox potential for eliminating ROS species, such as through the pools of antioxidants like ascorbic acid, glutathione, and α‐tocopherol through the biosynthesis of these compounds, as well as antioxidant enzymes such as superoxide dismutase, peroxidases, dehydroascorbate reductase, ascorbate oxidase, and catalases (Bode et al. 1995; Fang and Kao 2000; Wairich, Wang, et al. 2024). In a recent study, S‐nitrosoglutathione reductase (GSNOR) was reported to induce Fe toxicity tolerance in the root (Li et al. 2019). Like many abiotic and biotic stress regulators, WRKY transcription factors play a crucial role in ROS defence and Fe excess tolerance (Aung and Masuda 2020). Notably, OsWRKY76 acts as a negative regulator by suppressing Fe uptake genes and disrupting Fe homeostasis in rice (Mirza et al. 2023).

## 5 Coexpression of Molecular Components for Fe Homeostasis Regulation Under Fe Excess

**FIGURE 3:**
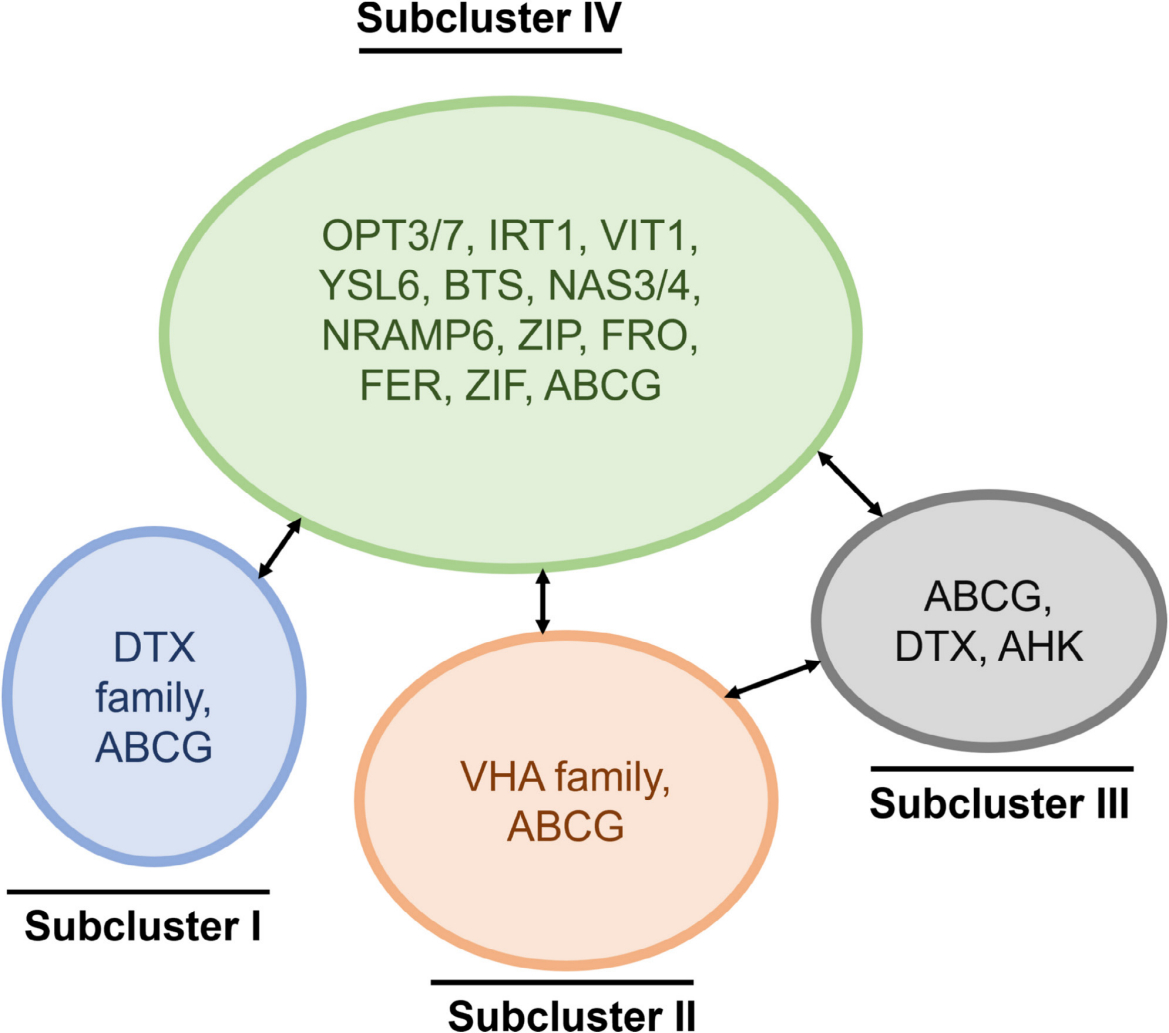
Summary of coexpression clusters of iron (Fe)‐excess‐responsive genes encoding indicated proteins. The indicated proteins are regulated at transcriptional level, and their functions depicted in Table S1. Genes of the four subclusters I to IV are shown in Figure S1 and Table S4.

Fe deficiency‐regulated genes involved in steering Fe homeostasis responses are largely co‐expressed with each other, which is due to their regulation by a common transcription factor cascade (Kobayashi et al. 2014; Schwarz and Bauer 2020). Lists of key regulatory genes involved in iron uptake, translocation, retention, and storage have been used in custom enrichment analyses using transcriptomic and proteomic studies, as well as microRNA analysis. Using the Fe homeostasis gene lists reported by Mai et al. (2023) and Wu et al. (2017) we generated a comprehensive list of Fe‐responsive genes in rice, providing a curated dataset for further functional characterization (Table S2). Refined co‐expression analysis within a group of Fe stress‐responsive genes, based on combined scores for all potential interactions, is a way to dissect the regulatory pathways and cross‐talks. Four sub‐groups of genes represent remarkable co‐expression subclusters (Figures 3 and S1, Table S3). Co‐expression subcluster I consists of genes encoding the DETOXIFICATION EFFLUX CARRIER (*DTX*) family proteins, which belong to the multidrug‐resistant transporter (MDRT) superfamily. DTX proteins play crucial roles in cellular protection by facilitating detoxification mechanisms, safeguarding cells and membrane‐bound organelles. The homologous rice gene *OsFRDL1* (Ferric Reductase Defective Like) has been characterized as a citrate transporter essential for the efficient translocation of Fe (Yokosho et al. 2009). Most DTX‐encoding genes and proteins have not yet been studied in rice. Fe excess‐regulated *DTX* genes may act in the transport of stress plant hormones like ABA, secondary metabolites produced under excess Fe or excess Fe‐binding compounds (Yokosho et al. 2009; Zhang et al. 2014; Ma et al. 2023; Figures 3 and S1, Table S4). Co‐expression subcluster II consists of vacuolar‐type (V‐type) H^+^‐ATPases (VHAs), localized in the vacuolar membrane and endomembrane compartments such as the trans‐Golgi network/early endosome (TGN/EE), which generate the electrochemical gradient for active transport, which may include transport processes relevant to Fe sequestration, metal ion transport, and DTX‐mediated transport (Figures 3 and S1, Table S4). Co‐expression subcluster III consists of ATP‐binding cassette (ABC) transporters, one of the largest and most evolutionarily conserved families of transporter proteins that use energy from ATP hydrolysis to move a wide range of molecules across membranes, including xenobiotics, hormones, sugars, amino acids, and metal ions (Figures 3 and S1, Table S4). In *Arabidopsis thaliana* , key ABCG (ATP‐binding cassette subfamily G) transporters such as ABCG36 and ABCG40 have been implicated in heavy metal resistance and cellular detoxification, highlighting their crucial role in protecting plants from metal toxicity (Dhara and Raichaudhuri 2021). The largest group, co‐expression subcluster IV, includes the components that play a crucial role in the various aspects of iron homeostasis as explained above for the Fe excess stress mitigation strategies according to categories I, II, and III (Figures 3 and S1, Table S4).

The co‐expression clusters of Fe excess‐regulated genes suggest the involvement of common cis‐ and trans‐regulatory factors in the iron excess response. A recent study by Kakei et al. (2021) employed bioinformatics and machine learning approaches to identify novel cis‐regulatory elements (CREs) associated with the Fe excess response in rice. They discovered 560 CRE candidates and 42 conserved sequences directly related to the Fe excess response across various rice tissues. Furthermore, the relationship between Fe toxicity and deficiency signaling is yet unclear. To maintain iron (Fe) homeostasis, plants must tightly regulate the transcriptional cascade that promotes Fe acquisition, especially under Fe excess conditions. This repression is mediated by Fe‐responsive E3 ubiquitin ligases such as HRZ1 (Hemerythrin E3 Ligases 1) and HRZ2, which target key transcription factors for degradation to suppress the Fe deficiency response (Long et al. 2010; Kobayashi et al. 2013; Li et al. 2021; Lichtblau et al. 2022). Recent advances have further underscored the role of bHLH subgroup IVc transcription factors, such as OsbHLH058, OsbHLH059, OsbHLH57, and OsbHLH60 in rice (Gao and Dubos 2021). These bHLH IVc transcription factors may interact with subgroup IVb member IRO3 (Basic Helix–Loop–Helix‐Type Transcription Factor; OsbHLH63) and OsbHLH62 (Wang et al. 2025), coordinating Fe uptake regulation and the fine‐tuning of Fe homeostasis gene expression, as seen in studies by Lichtblau et al. (2022) and Long et al. (2010). IRON MAN (IMA), also referred to as Fe‐uptake inducing peptides (FEPs), serves as short signaling molecules that mediate plant responses to Fe deficiency by counteracting HRZ and thereby enhancing the expression of Fe transport and regulatory genes, thereby facilitating Fe absorption and redistribution effectively (Li et al. 2021; Grillet et al. 2018; Lichtblau et al. 2022). In rice, the genes *OsIMA1* and *OsIMA2*, which encode IMA peptides, are strongly upregulated under Fe‐deficient conditions, reinforcing the iron uptake pathway and positively influencing overall Fe homeostasis (Grillet et al. 2018; Kobayashi et al. 2021; Li et al. 2021).

However, on the contrary, activation of Fe deficiency‐inducible genes was reported under Fe sufficiency in E3 ligase knock‐down rice varieties (Kobayashi et al. 2013; Hindt et al. 2017). These findings underscore the complex regulatory networks governing Fe uptake and distribution in rice, highlighting the importance of E3 ligases and transcription factors in balancing Fe homeostasis (Liang 2022). The functionality of HRZ1/2 E3 ligases in Fe excess in rice has clearly to be studied in more depth. These E3 ligases can bind Fe via their hemerythrin domains, which appears to influence their protein stability (Selote et al. 2015). Perhaps HRZ1/2 are Fe sensors to induce an immediate response after Fe supply (Hindt et al. 2017; Aung et al. 2018; Aung and Masuda 2020), a condition that has been experimentally applied in multiple Fe excess rice studies (Table S1).

## 6 Subcellular Localization of Key Regulatory Proteins for Controlling Fe Excess

**FIGURE 4:**
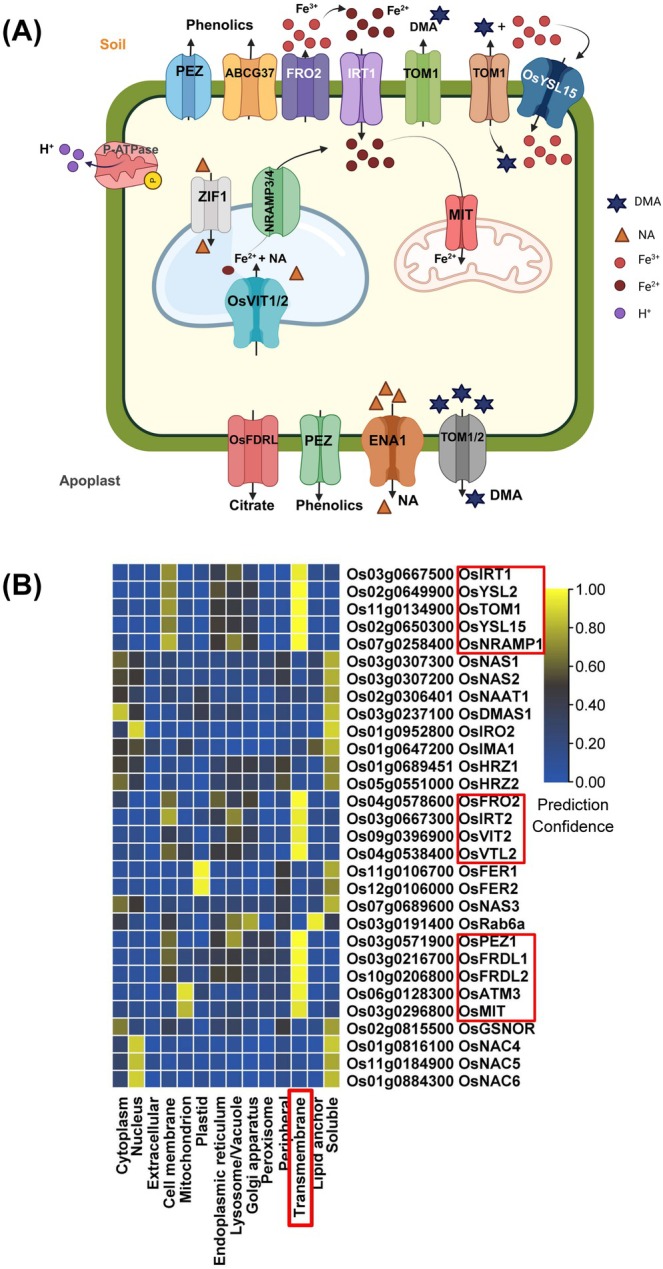
Subcellular location of transport proteins involved in iron (Fe) excess. (A) Schematic illustration of a cell with locations of trans‐membrane proteins in various compartment membranes regulated under Fe excess (Created with Biorender). (B) Heat map to illustrate predicted subcellular location of proteins encoded by the Fe excess‐responsive genes. Annotation for rice genes in heat map was done by referring to Arabidopsis as a reference. Plasma membrane components are boxed. The indicated protein functions are depicted in Table S1.

The activities of encoded proteins can be themselves controlled at post‐translational and protein level. An effective regulatory mechanism to control Fe homeostasis proteins under excess Fe is acting at subcellular localization to confer protein degradation. Hence, visual representation of the subcellular localization of proteins helps predict the molecular mechanisms that may govern protein abundance (Tables S3 and S5). Regulators such as the transcription factors and E3 ligases are expected to reside mostly in the nucleus or else the cytoplasm. Another group of proteins related to nicotianamine and phytosiderophore synthesis, like NAS1 (Nicotianamine Synthase), NAS2, NAAT1, DMAS1 as well as regulators like small protein or peptide IMA1, involved in categories I, II, and III are predicted to be localized in the cytoplasm. GSNOR is likewise reported to be localized in the cytoplasm. Other proteins are localized in plastids, such as FER proteins. Strategy III regulatory proteins were predicted to be localized at the mitochondrial membranes, such as MIT and ATM3. Very interestingly, based on their predicted subcellular location, many encoded proteins function in a membrane as transmembrane proteins, particularly of the plasma membrane, such as IRT1, YSL2 (Yellow Stripe1‐Like), YSL15, NRAMP1 (Natural Resistance‐Associated Macrophage Proteins), TOM1 (Transporter of Mugineic Acid Family Phytosiderophores), and FRO2 (Ferric‐Chelate Reductase Gene), the vacuolar tonoplast membrane, such as NRAMP3/4, VIT2 (Vacuolar Iron Transporter), VTL2, all involved in defense categories I and II (Figure 4). Upon shifts to excess Fe, protein degradation may be initiated to down‐regulate the abundance of proteins involved in Fe uptake from soil and the release of Fe from storage compartments. For plasma membrane proteins, this may involve phosphorylation and ubiquitination of intracellular protein portions, assembly of protein complexes, followed by endocytosis and vacuolar degradation, as reported for Arabidopsis IRT1, FRO2, and NRAMP1 proteins (Connolly et al. 2002; Connolly et al. 2003; Ivanov et al. 2014; Agorio et al. 2017; Dubeaux et al. 2018; Martín‐Barranco et al. 2020). Lipid peroxidation stress elicited through Fe^2+^ import across the plasma membrane may be reduced by lipophilic antioxidants like a‐tocopherol through the action of iron transporter‐binding lipid transfer proteins (Hornbergs et al. 2023). Hence, protein control in membrane compartments like the plasma membrane proteins, tonoplast, or other places is crucial for adjustments upon shifts to excess Fe, and specific proteolytic and vacuolar degradation pathways may be in place (Rodriguez‐Furlan et al. 2019). To date, there is no evidence whether any of these mechanisms play a role in the genetic adaptation of rice to Fe toxicity in acidic soils.

## 7 Meta‐Analysis of Differential Regulation Using Transcriptome Data for Fe Excess

We conducted an integrative meta‐analysis that consolidates RNA‐seq and microarray datasets from multiple independent studies to unravel the complexities of Fe homeostasis in rice under excess Fe conditions. By integrating differentially expressed genes from Finatto et al. (2015), Kar et al. (2021), Regon et al. (2022), and Wu et al. (2017), we established a comprehensive comparative framework that spans diverse genetic backgrounds, iron concentrations, treatment durations, and developmental stages (Table 1). This integrative approach overcomes the limitations of single‐variety studies, enhancing the robustness of Fe‐responsive gene identification and revealing fundamental mechanisms of Fe excess tolerance.

Our analysis identified key regulatory patterns across multiple datasets, reinforcing the critical roles of conserved Fe‐responsive genes. Wu et al. (2017) highlighted genes coding for *FER2* and *VIT2* as major contributors to Fe sequestration, while Kar et al. (2021) demonstrated a root–shoot coordination strategy, where shoots upregulate detoxification genes (encoding *HMA7, ATPases, ABCG*), and roots modulate Fe influx (coding for *MTP8, FER2, PDR12*). Regon et al. (2022) revealed a developmental shift in Fe homeostasis, with seedlings prioritizing Fe uptake and detoxification, whereas mature plants emphasize sequestration through genes encoding *MATE, ZIFL1* (Zinc‐Induced Facilitator Like 1), and *ABCG*. Finatto et al. (2015) further emphasized the role of genes coding for ABC transporters and PDR in stress adaptation, revealing trade‐offs between Fe regulation and plant growth.

**FIGURE 5:**
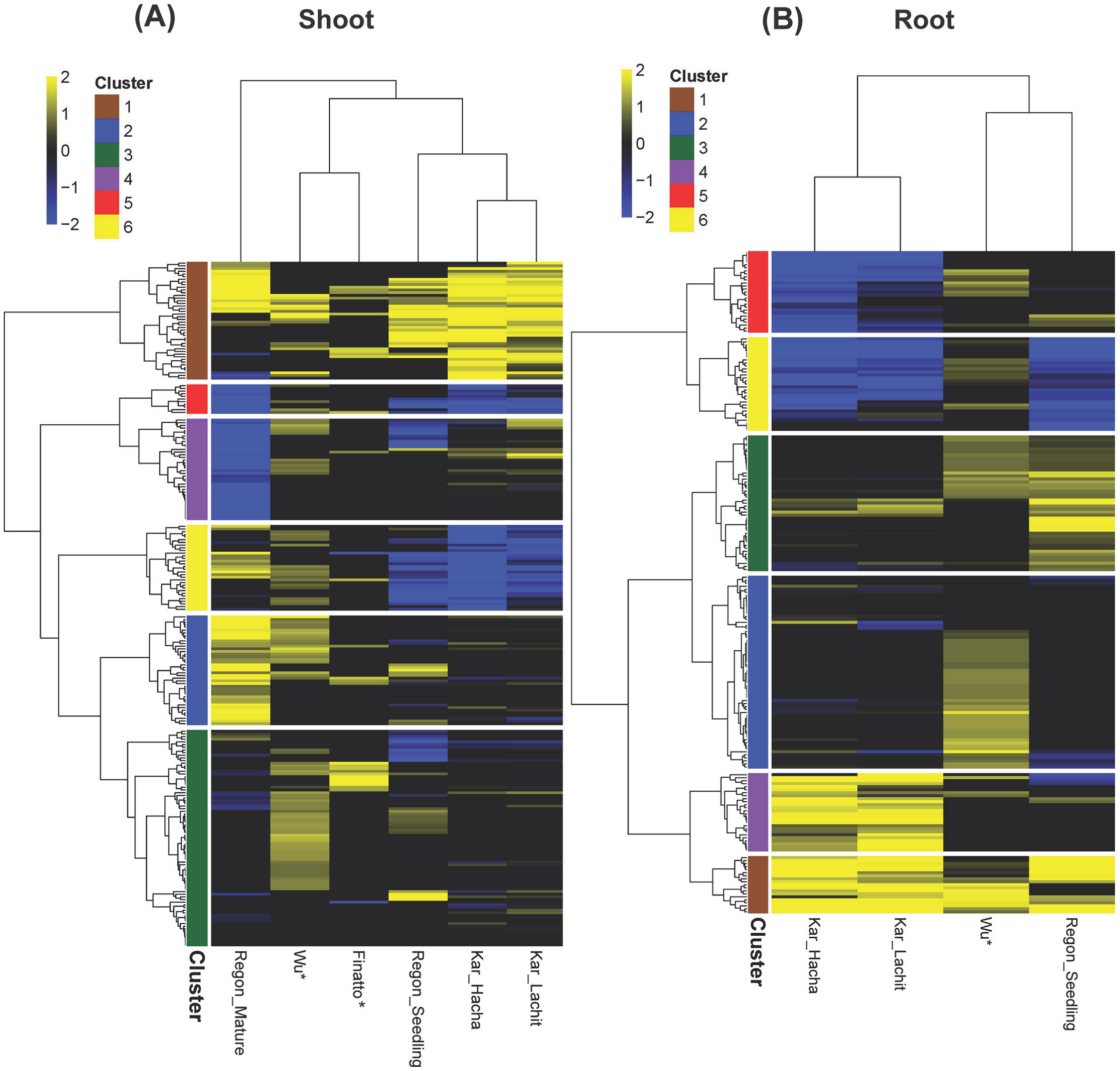
Hierarchical clustering and heat maps of differentially regulated genes under excess Fe identified in different Fe excess studies (Finatto et al. 2015; Wu et al. 2017; Kar et al. 2021; Regon et al. 2022). The analysis was conducted separately for (A) shoots and (B) roots. The genes are listed in Table S1; the genes within each cluster 1–6 are listed in Table S2.

**FIGURE 6:**
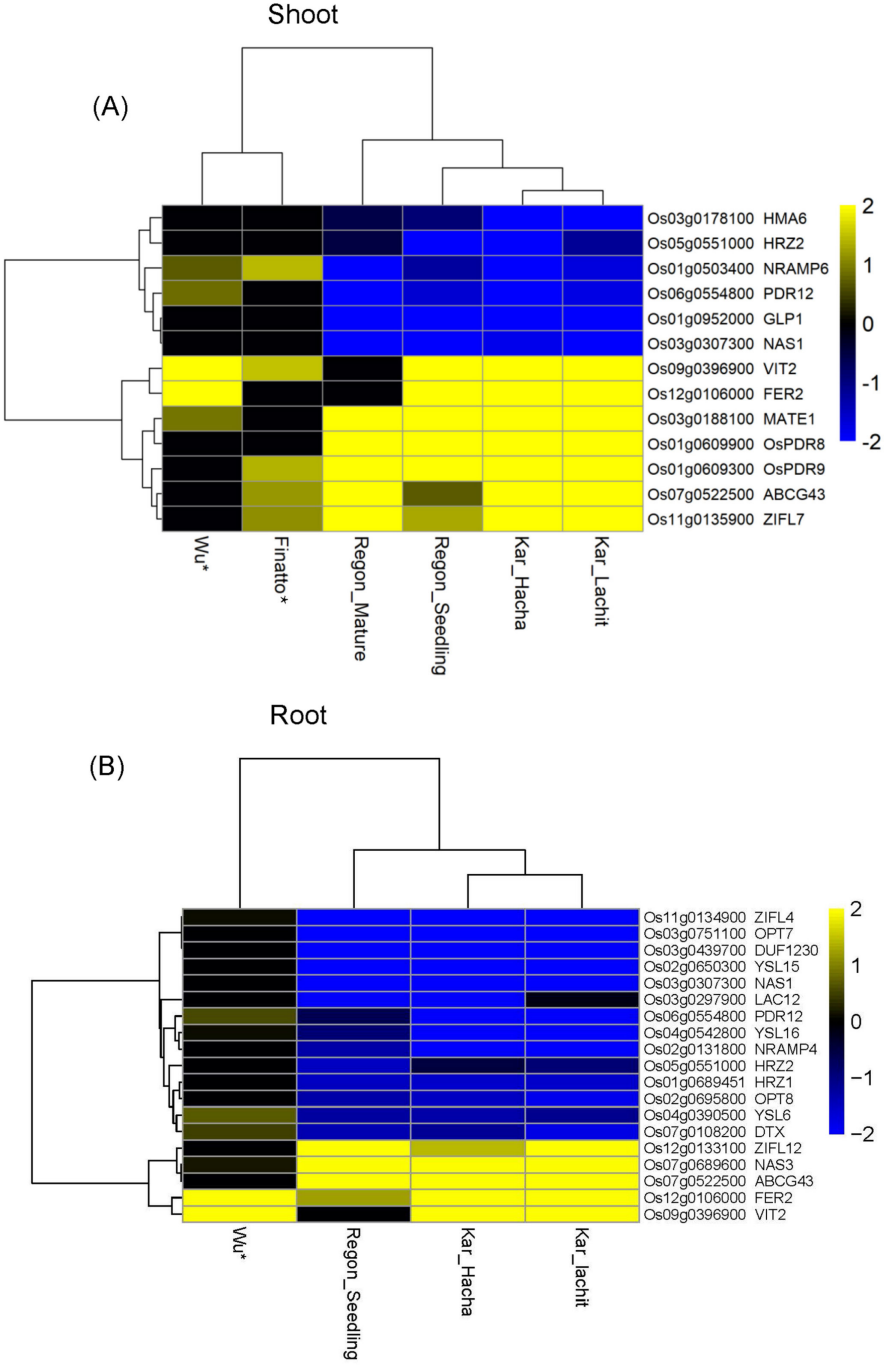
Hierarchical clustering and heat maps of iron (Fe)‐responsive genes that were found similarly regulated under Fe excess in root and shoot samples, based on the data sets by (Finatto et al. 2015; Wu et al. 2017; Kar et al. 2021; Regon et al. 2022). The indicated protein functions are depicted in (Table S1). Gene expression information is provided in Table S2.

A systematic cross‐study analysis enabled the identification of a core set of Fe‐regulated genes in both roots and shoots (Figures 5 and 6, Table S2). Notably, genes such as those encoding *HRZ1, NRAMP6, PDR12*, and *NAS1* were consistently downregulated across all datasets by Fe excess, suggesting a conserved function in limiting Fe uptake and facilitating exclusion from leaves. Conversely, genes encoding *VIT2, FER2*, and *ABCG43* were consistently upregulated under Fe toxicity, underscoring their essential roles in Fe sequestration, storage, and detoxification. The upregulation of VIT2 aligns with functional evidence showing that the double knockout of *OsVIT1* and *OsVIT2* compromises Fe tolerance, revealing a trade‐off between iron biofortification and excess Fe detoxification (Benato et al. 2025). These conserved regulatory mechanisms highlight potential conserved molecular markers for Fe toxicity stress, paving the way for future research into their transcriptional regulation.

Our findings underscore a development‐ and tissue‐specific regulatory network governing Fe homeostasis in rice. While seedlings rely on root‐driven Fe uptake control and shoot‐mediated sequestration, mature plants transition toward a regulated storage strategy to mitigate Fe toxicity. A root‐shoot coordination mechanism fine‐tunes Fe acquisition, detoxification, and oxidative stress responses, ensuring optimal Fe homeostasis under excess Fe conditions. These insights offer a valuable foundation for future genetic and biotechnological strategies aimed at enhancing Fe toxicity tolerance in rice.

## 8 Discussion and Future Prospectives

This review serves as a critical bridge between existing studies on Fe excess in rice by synthesizing transcriptomic data from multiple independent research efforts, exploiting different types of lowland rice germplasm. The meta‐analysis approach specifically bridges gaps by: (1) Consolidating gene expression data across different experimental conditions and rice varieties. (2) Identifying common molecular pathways and regulatory networks that transcend individual studies. (3) Highlighting consistent patterns of differential gene expression that may represent core adaptive responses. (4) Connecting phenotypic observations with underlying molecular mechanisms. (5) Providing a framework for understanding how various environmental factors interact with genetic components. This integrative approach validates findings across multiple studies while also revealing novel insights that would not be apparent from any single investigation, thereby establishing a more comprehensive understanding of Fe excess responses in rice.

This review has uncovered substantial gaps in our understanding and management of Fe excess tolerance mechanisms, which, if addressed, could significantly enhance our ability to improve crop productivity and sustainability in iron‐toxic environments through plant biotechnological interventions. Key knowledge gaps in Fe toxicity research include the lack of comparative studies between tolerant and susceptible rice lines, limited genetic diversity in current panels, and insufficient control of experimental conditions across developmental stages. Moreover, protein‐level regulation and functional validation of candidate genes remain underexplored, highlighting the need for integrative, cell‐biological, and genetic approaches to better understand and improve Fe excess tolerance in rice.

With such knowledge in hand, future plant breeding studies can be pinpointed at enhancing the performance of susceptible rice lines, particularly for growth in acidic soils. Future research should consider advanced genome‐editing strategies, such as knock‐in and knock‐out approaches, to directly modify genetic traits in these varieties. Tailoring such interventions to improve substrate tolerance will ultimately pave the way for rice varieties that are not only more resilient to Fe toxicity but also better adapted to a range of soil conditions. Thus, an integrative, multidisciplinary approach can help bridge the identified knowledge gaps for a transformative understanding of Fe excess tolerance in rice and potentially other crops cultivated for thriving in iron‐toxic and acidic soils, which is imperative for ensuring global food security amidst ever‐changing environmental conditions.

## 9 Open Questions and Knowledge Gaps

One major shortcoming is the lack of a direct comparative study between identified tolerant and susceptible rice lines. This gap underlines the need for comprehensive genome‐wide association studies (GWAS) that focus on Fe excess adaptation.

Future work should push beyond conventional panels by incorporating a greater biodiversity of genetic material such as African and South American rice lines, as well as wild rice species, which are to date understudied. For example, Wairich et al. (2021) included *Oryza meridionalis*, a wild Australian rice species, to explore transcriptional responses to Fe excess. Expanding the panel in this manner may unearth novel alleles responsible for Fe tolerance that have been overlooked in traditional studies.

Current studies generally suffer from a lack of systematic comparisons of responses to Fe excess under varying experimental conditions. A carefully controlled study, examining different plant developmental stages, varying Fe supply concentrations, and distinct durations of exposure, would provide a more nuanced understanding of the physiological and developmental dynamics under stress. Such an approach would help isolate the specific responses attributable to developmental stage versus environmental stimuli, thereby refining both experimental methods and data interpretation.

A further notable deficiency in the field is the scarcity of studies addressing protein‐level regulation during Fe excess. The incorporation of cell biological techniques—including subcellular localization analyses, quantitative immunoblotting, and protein interaction and protein degradation studies—is urgently needed. This should focus particularly on understanding the control of plasma membrane proteins via the vesicular trafficking system under conditions of excess Fe. Addressing this gap will significantly enhance our understanding of post‐translational modifications and the dynamic regulation of key stress response proteins.

There is a critical shortfall in the functional validation of individual genes and proteins implicated in Fe excess tolerance. Future efforts should catalyse genetic studies that manipulate the activity of these genes to confirm their roles. Integrating these insights with the known regulators of the Fe acquisition response under excess Fe conditions will allow for a more robust and comprehensive model of Fe homeostasis. For a start, the identified conserved marker genes may be functionally investigated in the first place.

## Author Contributions

D.G. analyzed the data and wrote the paper. P.B. and S.K.P. conceptualized, supervised, and improved the paper. All authors have read and approved the final manuscript.

## Supporting information


**Figure S1:** Co‐expression analysis of excess iron (Fe)‐responsive genes to predict their interaction with others to improve plant tolerance to stress.

**Table S1:** List of excessive iron (Fe)‐responsive genes involved in different strategies as plant's defence system.

**Table S2:** List of iron (Fe)‐responsive genes in rice and their gene expression data, differentially expressed Fe‐responsive genes and their subsets based on different data set analyses in root and shoot tissues.

**Table S3:** List of genes in the coexpression network analysis based on their combined score and prediction of subcellular localization of listed genes predicted using DeepLoc‐2.0.

**Table S4:** Summary of protein functions grouped in gene coexpression network.

**Table S5:** Subcellular localization, signal, and membrane types of Iron (Fe) excess‐responsive genes.

## Data Availability

The data that support the findings of this study are available from the corresponding author upon request.

## References

[ppl70473-bib-0001] Agegnehu, G. , T. Amede , T. Erkossa , et al. 2021. “Extent and Management of Acid Soils for Sustainable Crop Production System in the Tropical Agroecosystems: A Review.” Acta Agriculturae Scandinavica Section B Soil and Plant Science 71, no. 9: 852–869.

[ppl70473-bib-0002] Agorio, A. , J. Giraudat , M. W. Bianchi , et al. 2017. “Phosphatidylinositol 3‐Phosphate–Binding Protein AtPH1 Controls the Localization of the Metal Transporter NRAMP1 in Arabidopsis.” Proceedings of the National Academy of Sciences of the United States of America 114, no. 16: E3354–E3363. 28373552 10.1073/pnas.1702975114 PMC5402440

[ppl70473-bib-0004] Aung, M. S. , T. Kobayashi , H. Masuda , and N. K. Nishizawa . 2018. “Rice HRZ Ubiquitin Ligases Are Crucial for the Response to Excess Iron.” Physiologia Plantarum 163, no. 3: 282–296. 10.1111/ppl.12698 29655221

[ppl70473-bib-0005] Aung, M. S. , and H. Masuda . 2020. “How Does Rice Defend Against Excess Iron?: Physiological and Molecular Mechanisms.” Frontiers in Plant Science 11: 1102. 32849682 10.3389/fpls.2020.01102 PMC7426474

[ppl70473-bib-0008] Bashir, K. , K. Hanada , M. Shimizu , M. Seki , H. Nakanishi , and N. K. Nishizawa . 2014. “Transcriptomic Analysis of Rice in Response to Iron Deficiency and Excess.” Rice 7: 1–15. 26224551 10.1186/s12284-014-0018-1 PMC4884027

[ppl70473-bib-0009] Bashir, K. , Y. Ishimaru , H. Shimo , et al. 2011. “The Rice Mitochondrial Iron Transporter Is Essential for Plant Growth.” Nature Communications 2, no. 1: 322. 10.1038/ncomms1326 PMC3113228 21610725

[ppl70473-bib-0013] Becker, M. , and F. Asch . 2005. “Iron Toxicity in Rice—Conditions and Management Concepts.” Journal of Plant Nutrition and Soil Science 168, no. 4: 558–573.

[ppl70473-bib-0014] Benato, B. D. , A. G. S. Rativa , R. V. Olsson , et al. 2025. “Double Knockout of Rice OsVIT1 and OsVIT2 Genes Reveals a Trade‐Off Between Iron Biofortification and Iron Excess Tolerance.” bioRxiv: 2025‐06. 10.1093/jxb/eraf505 41257534

[ppl70473-bib-0015] Bencze, K. Z. , K. C. Kondapalli , J. D. Cook , et al. 2006. “The Structure and Function of Frataxin.” Critical Reviews in Biochemistry and Molecular Biology 41, no. 5: 269–291. 16911956 10.1080/10409230600846058 PMC2859089

[ppl70473-bib-0017] Bode, K. , O. Döring , S. Lüthje , H. U. Neue , and M. Böttger . 1995. “The Role of Active Oxygen in Iron Tolerance of Rice (Oryza Sauva L.).” Protoplasma 184: 249–255.

[ppl70473-bib-0018] Bojórquez‐Quintal, E. , C. Escalante‐Magaña , I. Echevarría‐Machado , and M. Martínez‐Estévez . 2017. “Aluminum, a Friend or Foe of Higher Plants in Acid Soils.” Frontiers in Plant Science 8: 1767. 29075280 10.3389/fpls.2017.01767 PMC5643487

[ppl70473-bib-0021] Chen, S. , R. Sánchez‐Fernández , E. R. Lyver , A. Dancis , and P. A. Rea . 2007. “Functional Characterization of AtATM1, AtATM2, and AtATM3, a Subfamily of Arabidopsis Half‐Molecule ATP‐Binding Cassette Transporters Implicated in Iron Homeostasis.” Journal of Biological Chemistry 282, no. 29: 21561–21571. 17517886 10.1074/jbc.M702383200

[ppl70473-bib-0024] Connolly, E. L. , N. H. Campbell , N. Grotz , C. L. Prichard , and M. L. Guerinot . 2003. “Overexpression of the FRO2 Ferric Chelate Reductase Confers Tolerance to Growth on Low Iron and Uncovers Posttranscriptional Control.” Plant Physiology 133, no. 3: 1102–1110. 14526117 10.1104/pp.103.025122 PMC281606

[ppl70473-bib-0025] Connolly, E. L. , J. P. Fett , and M. L. Guerinot . 2002. “Expression of the IRT1 Metal Transporter Is Controlled by Metals at the Levels of Transcript and Protein Accumulation.” Plant Cell 14, no. 6: 1347–1357. 12084831 10.1105/tpc.001263 PMC150784

[ppl70473-bib-0028] Dhara, A. , and A. Raichaudhuri . 2021. “ABCG Transporter Proteins With Beneficial Activity on Plants.” Phytochemistry 184: 112663. 33550197 10.1016/j.phytochem.2021.112663

[ppl70473-bib-0030] Dobermann, A. 2000. “Rice: Nutrient Disorders & Nutrient Management.” International Rice Research Institute.

[ppl70473-bib-0031] Dubeaux, G. , J. Neveu , E. Zelazny , and G. Vert . 2018. “Metal Sensing by the IRT1 Transporter‐Receptor Orchestrates Its Own Degradation and Plant Metal Nutrition.” Molecular Cell 69, no. 6: 953–964. 29547723 10.1016/j.molcel.2018.02.009

[ppl70473-bib-0033] Fageria, N. K. , and A. S. Nascente . 2014. “Management of Soil Acidity of South American Soils for Sustainable Crop Production.” Advances in Agronomy 128: 221–275.

[ppl70473-bib-0034] Fang, W. C. , and C. H. Kao . 2000. “Enhanced Peroxidase Activity in Rice Leaves in Response to Excess Iron, Copper and Zinc.” Plant Science 158, no. 1–2: 71–76. 10996246 10.1016/s0168-9452(00)00307-1

[ppl70473-bib-0036] Finatto, T. , A. C. de Oliveira , C. Chaparro , et al. 2015. “Abiotic Stress and Genome Dynamics: Specific Genes and Transposable Elements Response to Iron Excess in Rice.” Rice 8: 1–18. 25844118 10.1186/s12284-015-0045-6 PMC4385019

[ppl70473-bib-0038] Gao, F. , and C. Dubos . 2021. “Transcriptional Integration of Plant Responses to Iron Availability.” Journal of Experimental Botany 72, no. 6: 2056–2070. 33246334 10.1093/jxb/eraa556

[ppl70473-bib-0040] Grillet, L. , P. Lan , W. Li , G. Mokkapati , and W. Schmidt . 2018. “IRON MAN Is a Ubiquitous Family of Peptides That Control Iron Transport in Plants.” Nature Plants 4, no. 11: 953–963. 30323182 10.1038/s41477-018-0266-y

[ppl70473-bib-0122] Hindt, M. N. , G. Z. Akmakjian , K. L. Pivarski , et al. 2017. “BRUTUS and its Paralogs, BTS LIKE1 and BTS LIKE2, Encode Important Negative Regulators of the Iron Deficiency Response in Arabidopsis Thaliana.” Metallomics 9, no. 7: 876–890. 28620661 10.1039/c7mt00152e PMC5558852

[ppl70473-bib-0123] Hornbergs, J. , K. Montag , J. Loschwitz , et al. 2023. “SEC14‐GOLD protein PATELLIN2 Binds IRON‐REGULATED TRANSPORTER1 Linking Root Iron Uptake to Vitamin E.” Plant Physiology 192, no. 1: 504–526. 36493393 10.1093/plphys/kiac563 PMC10152663

[ppl70473-bib-0047] Hossain, M. A. , P. Piyatida , J. A. T. da Silva , and M. Fujita . 2012. “Molecular Mechanism of Heavy Metal Toxicity and Tolerance in Plants: Central Role of Glutathione in Detoxification of Reactive Oxygen Species and Methylglyoxal and in Heavy Metal Chelation.” Journal of Botany 2012, no. 1: 872875.

[ppl70473-bib-0048] Inoue, H. , K. Higuchi , M. Takahashi , H. Nakanishi , S. Mori , and N. K. Nishizawa . 2003. “Three Rice Nicotianamine Synthase Genes, OsNAS1, OsNAS2, and OsNAS3 Are Expressed in Cells Involved in Long‐Distance Transport of Iron and Differentially Regulated by Iron.” Plant Journal 36, no. 3: 366–381. 10.1046/j.1365-313x.2003.01878.x 14617093

[ppl70473-bib-0049] Ivanov, R. , T. Brumbarova , A. Blum , A. M. Jantke , C. Fink‐Straube , and P. Bauer . 2014. “SORTING NEXIN1 Is Required for Modulating the Trafficking and Stability of the Arabidopsis IRON‐REGULATED TRANSPORTER1.” Plant Cell 26, no. 3: 1294–1307. 24596241 10.1105/tpc.113.116244 PMC4001385

[ppl70473-bib-0050] Kakei, Y. , H. Masuda , N. K. Nishizawa , H. Hattori , and M. S. Aung . 2021. “Elucidation of Novel Cis‐Regulatory Elements and Promoter Structures Involved in Iron Excess Response Mechanisms in Rice Using a Bioinformatics Approach.” Frontiers in Plant Science 12: 660303. 34149757 10.3389/fpls.2021.660303 PMC8207140

[ppl70473-bib-0052] Kar, S. , H. J. Mai , H. Khalouf , et al. 2021. “Comparative Transcriptomics of Lowland Rice Varieties Uncovers Novel Candidate Genes for Adaptive Iron Excess Tolerance.” Plant and Cell Physiology 62, no. 4: 624–640. 33561287 10.1093/pcp/pcab018 PMC8462385

[ppl70473-bib-0121] Kobayashi, T. , A. J. Nagano , and N. K. Nishizawa . 2021. “Iron Deficiency‐Inducible Peptide‐Coding Genes OsIMA1 and OsIMA2 Positively Regulate a Major Pathway of Iron Uptake and Translocation in Rice.” Journal of Experimental Botany 72, no. 6: 2196–2211. 33206982 10.1093/jxb/eraa546

[ppl70473-bib-0055] Kobayashi, T. , S. Nagasaka , T. Senoura , R. N. Itai , H. Nakanishi , and N. K. Nishizawa . 2013. “Iron‐Binding Haemerythrin RING Ubiquitin Ligases Regulate Plant Iron Responses and Accumulation.” Nature Communications 4, no. 1: 2792. 10.1038/ncomms3792 PMC3905729 24253678

[ppl70473-bib-0056] Kobayashi, T. , R. Nakanishi Itai , and N. K. Nishizawa . 2014. “Iron Deficiency Responses in Rice Roots.” Rice 7: 1–11. 26224556 10.1186/s12284-014-0027-0 PMC4884003

[ppl70473-bib-0060] Kushnir, S. , E. Babiychuk , S. Storozhenko , et al. 2001. “A Mutation of the Mitochondrial ABC Transporter Sta1 Leads to Dwarfism and Chlorosis in the Arabidopsis Mutant Starik.” Plant Cell 13, no. 1: 89–100. 11158531 10.1105/tpc.13.1.89 PMC102216

[ppl70473-bib-0061] Le, C. T. T. , T. Brumbarova , and P. Bauer . 2019. “The Interplay of ROS and Iron Signaling.” In Redox Homeostasis in Plants: From Signalling to Stress Tolerance, 43. Springer.

[ppl70473-bib-0062] Li, B. , L. Sun , J. Huang , et al. 2019. “GSNOR Provides Plant Tolerance to Iron Toxicity via Preventing Iron‐Dependent Nitrosative and Oxidative Cytotoxicity.” Nature Communications 10, no. 1: 3896. 10.1038/s41467-019-11892-5 PMC6715714 31467270

[ppl70473-bib-0064] Li, Y. , C. K. Lu , C. Y. Li , et al. 2021. “IRON MAN Interacts With BRUTUS to Maintain Iron Homeostasis in Arabidopsis.” Proceedings of the National Academy of Sciences of the United States of America 118, no. 39: e2109063118. 34548401 10.1073/pnas.2109063118 PMC8488653

[ppl70473-bib-0066] Liang, G. 2022. “Iron Uptake, Signaling, and Sensing in Plants.” Plant Communications 3, no. 5: 100349. 35706354 10.1016/j.xplc.2022.100349 PMC9483112

[ppl70473-bib-0067] Lichtblau, D. M. , B. Schwarz , D. Baby , C. Endres , C. Sieberg , and P. Bauer . 2022. “The Iron Deficiency‐Regulated Small Protein Effector FEP3/IRON MAN1 Modulates Interaction of BRUTUS‐LIKE1 With bHLH Subgroup IVc and POPEYE Transcription Factors.” Frontiers in Plant Science 13: 930049. 35755670 10.3389/fpls.2022.930049 PMC9226616

[ppl70473-bib-0068] Long, T. A. , H. Tsukagoshi , W. Busch , B. Lahner , D. E. Salt , and P. N. Benfey . 2010. “The bHLH Transcription Factor POPEYE Regulates Response to Iron Deficiency in Arabidopsis Roots.” Plant Cell 22, no. 7: 2219–2236. 20675571 10.1105/tpc.110.074096 PMC2929094

[ppl70473-bib-0069] López‐Millán, A. F. , D. Duy , and K. Philippar . 2016. “Chloroplast Iron Transport Proteins–Function and Impact on Plant Physiology.” Frontiers in Plant Science 7: 178. 27014281 10.3389/fpls.2016.00178 PMC4780311

[ppl70473-bib-0070] Ma, Y. , D. Li , Y. Zhong , et al. 2023. “Vacuolar MATE/DTX Protein‐Mediated Cucurbitacin C Transport Is Co‐Regulated With Bitterness Biosynthesis in Cucumber.” New Phytologist 238, no. 3: 995–1003. 36732026 10.1111/nph.18786

[ppl70473-bib-0071] Mai, H. J. , D. Baby , and P. Bauer . 2023. “Black Sheep, Dark Horses, and Colorful Dogs: A Review on the Current State of the Gene Ontology With Respect to Iron Homeostasis in *Arabidopsis Thaliana* .” Frontiers in Plant Science 14: 1204723. 37554559 10.3389/fpls.2023.1204723 PMC10406446

[ppl70473-bib-0072] Martín‐Barranco, A. , J. Spielmann , G. Dubeaux , G. Vert , and E. Zelazny . 2020. “Dynamic Control of the High‐Affinity Iron Uptake Complex in Root Epidermal Cells.” Plant Physiology 184, no. 3: 1236–1250. 32873629 10.1104/pp.20.00234 PMC7608170

[ppl70473-bib-0073] Mirza, Z. , S. Jonwal , H. Saini , A. K. Sinha , and M. Gupta . 2023. “Unraveling the Molecular Aspects of Iron‐Mediated OsWRKY76 Signaling Under Arsenic Stress in Rice.” Plant Physiology and Biochemistry 204: 108136. 37897891 10.1016/j.plaphy.2023.108136

[ppl70473-bib-0074] Mishra, A. , R. K. Nayak , G. Chander , M. Reddy , and P. Choudhari . 2020. “Management of Acidic Soils.” In Mapping the Nutrient Status of Odisha’s Soils, 1–55. ICRISAT.

[ppl70473-bib-0076] Morrissey, J. , and M. L. Guerinot . 2009. “Iron Uptake and Transport in Plants: The Good, the Bad, and the Ionome.” Chemical Reviews 109, no. 10: 4553–4567. 19754138 10.1021/cr900112r PMC2764373

[ppl70473-bib-0080] Ngoune Tandzi, L. , C. S. Mutengwa , E. L. M. Ngonkeu , and V. Gracen . 2018. “Breeding Maize for Tolerance to Acidic Soils: A Review.” Agronomy 8, no. 6: 84.

[ppl70473-bib-0088] Quinet, M. , D. Vromman , A. Clippe , et al. 2012. “Combined Transcriptomic and Physiological Approaches Reveal Strong Differences Between Short‐and Long‐Term Response of Rice (*Oryza sativa*) to Iron Toxicity.” Plant, Cell & Environment 35, no. 10: 1837–1859. 10.1111/j.1365-3040.2012.02521.x 22506799

[ppl70473-bib-0089] Rahman, S. U. , J. C. Han , M. Ahmad , et al. 2024. “Aluminum Phytotoxicity in Acidic Environments: A Comprehensive Review of Plant Tolerance and Adaptation Strategies.” Ecotoxicology and Environmental Safety 269: 115791. 38070417 10.1016/j.ecoenv.2023.115791

[ppl70473-bib-0091] Regon, P. , S. Dey , M. Rehman , et al. 2022. “Transcriptomic Analysis Revealed Reactive Oxygen Species Scavenging Mechanisms Associated With Ferrous Iron Toxicity in Aromatic Keteki Joha Rice.” Frontiers in Plant Science 13: 798580. 35283928 10.3389/fpls.2022.798580 PMC8913046

[ppl70473-bib-0094] Rodriguez‐Furlan, C. , E. A. Minina , and G. R. Hicks . 2019. “Remove, Recycle, Degrade: Regulating Plasma Membrane Protein Accumulation.” Plant Cell 31, no. 12: 2833–2854. 31628169 10.1105/tpc.19.00433 PMC6925004

[ppl70473-bib-0097] Rout, G. R. , and S. Sahoo . 2015. “Role of Iron in Plant Growth and Metabolism.” Reviews in Agricultural Science 3: 1–24.

[ppl70473-bib-0098] Schwarz, B. , and P. Bauer . 2020. “FIT, a Regulatory Hub for Iron Deficiency and Stress Signaling in Roots, and FIT‐Dependent And‐Independent Gene Signatures.” Journal of Experimental Botany 71, no. 5: 1694–1705. 31922570 10.1093/jxb/eraa012 PMC7067300

[ppl70473-bib-0099] Selote, D. , R. Samira , A. Matthiadis , J. W. Gillikin , and T. A. Long . 2015. “Iron‐Binding E3 Ligase Mediates Iron Response in Plants by Targeting Basic Helix‐Loop‐Helix Transcription Factors.” Plant Physiology 167, no. 1: 273–286. 25452667 10.1104/pp.114.250837 PMC4281009

[ppl70473-bib-0101] Stein, R. J. , G. L. Duarte , L. Scheunemann , et al. 2019. “Genotype Variation in Rice (*Oryza sativa* L.) Tolerance to Fe Toxicity Might Be Linked to Root Cell Wall Lignification.” Frontiers in Plant Science 10: 746. 31244872 10.3389/fpls.2019.00746 PMC6581717

[ppl70473-bib-0107] Vose, P. B. 1982. “Iron Nutrition in Plants: A World Overview.” Journal of Plant Nutrition 5, no. 4–7: 233–249.

[ppl70473-bib-0108] Wairich, A. , M. S. Aung , F. K. Ricachenevsky , and H. Masuda . 2024. “You Can't Always Get as Much Iron as You Want: How Rice Plants Deal With Excess of an Essential Nutrient.” Frontiers in Plant Science 15: 1381856. 39100081 10.3389/fpls.2024.1381856 PMC11294178

[ppl70473-bib-0109] Wairich, A. , B. H. N. De Oliveira , L. B. Wu , et al. 2021. “Chromosomal Introgressions From Oryza Meridionalis Into Domesticated Rice *Oryza sativa* Result in Iron Tolerance.” Journal of Experimental Botany 72: 2242–2259. 10.1093/jxb/eraa461. 33035327

[ppl70473-bib-0110] Wairich, A. , Y. Wang , B. T. Werner , Y. Vaziritabar , M. Frei , and L. B. Wu . 2024. “The Role of Ascorbate Redox Turnover in Iron Toxicity Tolerance.” Plant Physiology and Biochemistry 215: 109045. 39154421 10.1016/j.plaphy.2024.109045

[ppl70473-bib-0111] Wang, W. , F. He , H. Zhang , et al. 2025. “OsbHLH062 Regulates Iron Homeostasis by Inhibiting Iron Deficiency Responses in Rice.” aBIOTECH 6: 1–231. 40641637 10.1007/s42994-025-00203-w PMC12238704

[ppl70473-bib-0114] Wu, L. B. , Y. Ueda , S. K. Lai , and M. Frei . 2017. “Shoot Tolerance Mechanisms to Iron Toxicity in Rice ( *Oryza sativa* L.).” Plant, Cell & Environment 40, no. 4: 570–584. 10.1111/pce.12733 26991510

[ppl70473-bib-0117] Yokosho, K. , N. Yamaji , D. Ueno , N. Mitani , and J. F. Ma . 2009. “OsFRDL1 Is a Citrate Transporter Required for Efficient Translocation of Iron in Rice.” Plant Physiology 149, no. 1: 297–305. 19011004 10.1104/pp.108.128132 PMC2613705

[ppl70473-bib-0119] Zhang, H. , H. Zhu , Y. Pan , Y. Yu , S. Luan , and L. Li . 2014. “A DTX/MATE‐Type Transporter Facilitates Abscisic Acid Efflux and Modulates ABA Sensitivity and Drought Tolerance in Arabidopsis.” Molecular Plant 7, no. 10: 1522–1532. 24851876 10.1093/mp/ssu063

[ppl70473-bib-0120] Zhang, Y. , Y. H. Xu , H. Y. Yi , and J. M. Gong . 2012. “Vacuolar Membrane Transporters OsVIT1 and OsVIT2 Modulate Iron Translocation Between Flag Leaves and Seeds in Rice.” Plant Journal 72, no. 3: 400–410. https://www.fas.usda.gov/data/production/commodity/0422110; https://www.statista.com/statistics/255977/total‐global‐rice. 10.1111/j.1365-313X.2012.05088.x 22731699

