# Supplementary material for: Meta‐Analysis of Iron Excess Stress in Rice: Genes and Mechanisms of Tolerance to Acidic Soil
Source: Physiol Plant. 2025 Aug 27;177(5):e70473. doi: 10.1111/ppl.70473 (PMC12391641; doi:10.1111/ppl.70473)
Supplement: Supplementary file 1 — Figure S1: Co‐expression analysis of excess iron (Fe)‐responsive genes to predict their interaction with others to improve plant tolerance to stress. [file PPL-177-e70473-s005.pdf]

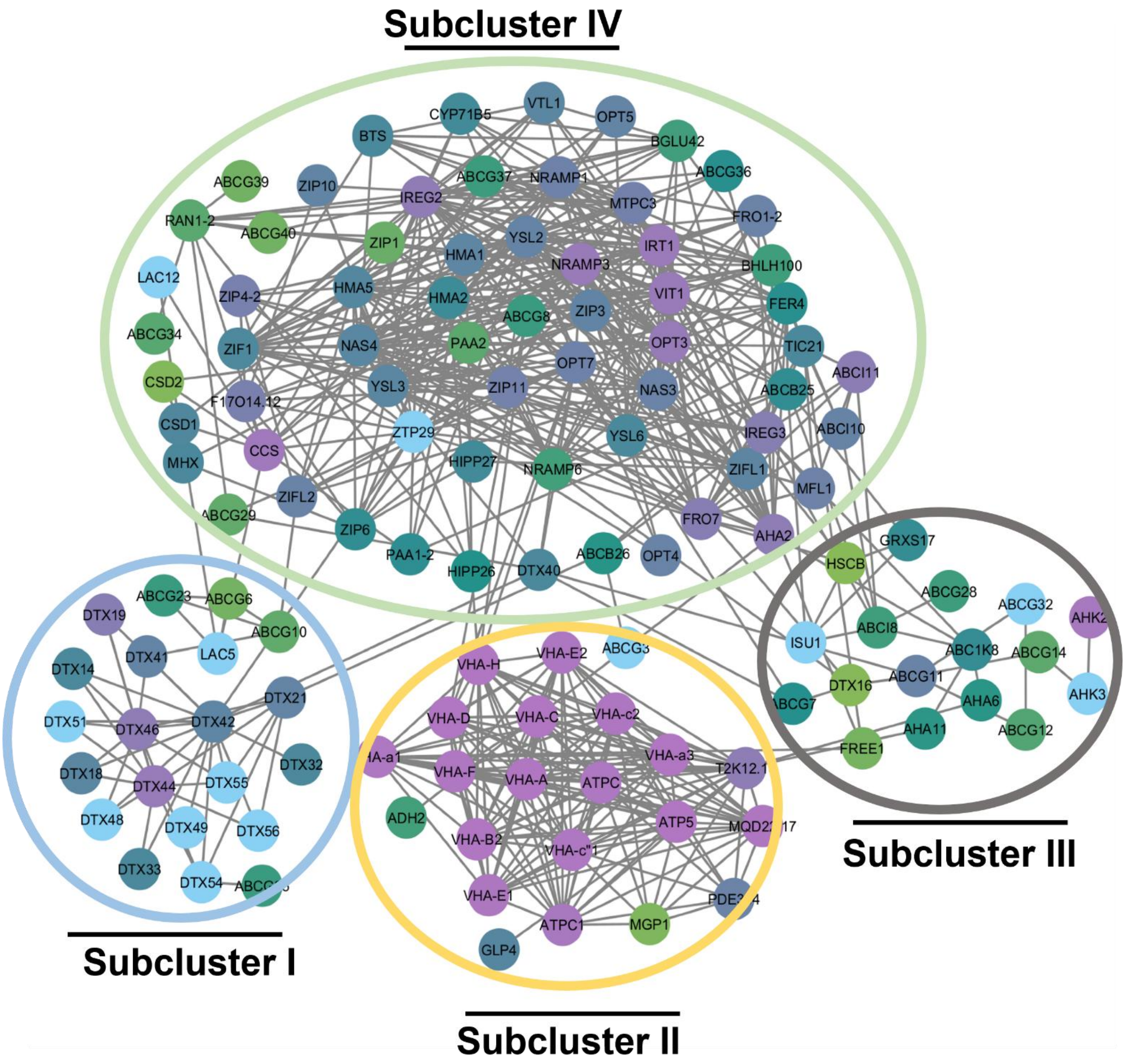
Subcluster IV
Subcluster I
Subcluster II
Subcluster III
VTL1
CYP71B5
OPT5
BTS
BGLU42
ABCG39
ZIP10
ABCG37
NRAMP1
ABCG36
IREG2
MTPC3
RAN1-2
FRO1-2
ABCG40
ZIP1
YSL2
IRT1
LAC12
HMA1
NRAMP3
BHLH100
ZIP4-2
HMA5
HMA2
VIT1
FER4
ABCG34
ABCG8
ZIP3
ZIF1
NAS4
PAA2
OPT3
TIC21
OPT7
ABCI11
CSD2
YSL3
ZIP11
NAS3
ABCB25
F17O14.12
CSD1
ZTP29
YSL6
IREG3
ABCI10
CCS
MHX
HIPP27
NRAMP6
ZIFL1
MFL1
ZIFL2
ABCG29
FRO7
ZIP6
AHA2
GRXS17
PAA1-2
ABCB26
OPT4
HIPP26
DTX40
HSCB
ABCG28
ABCG32
ABCG23
ABCG6
DTX19
AHK2
ABCI8
ABCG10
ISU1
ABC1K8
ABCG14
DTX41
LAC5
VHA-E2
ABCG3
VHA-H
DTX14
DTX21
DTX16
ABCG11
AHK3
AHA6
DTX51
DTX46
DTX42
VHA-D
VHA-C
VHA-c2
ABCG7
VHA-a3
DTX32
AHA11
ABCG12
FREE1
VHA-F
VHA-A
ATPC
T2K12.1
DTX18
DTX44
DTX55
ADH2
ATP5
DTX48
DTX49
DTX56
VHA-B2
VHA-c''1
DTX33
DTX54
VHA-E1
ATPC1
MGP1
GLP4

Figure S1- Co-expression analysis of excess iron (Fe)-responsive genes to predict their interaction with others to improve plant tolerance to stress. Their depicted functions are found in (Box 4; Table S1).
