# Supplementary material for: Meta‐Analysis of Iron Excess Stress in Rice: Genes and Mechanisms of Tolerance to Acidic Soil
Source: Physiol Plant. 2025 Aug 27;177(5):e70473. doi: 10.1111/ppl.70473 (PMC12391641; doi:10.1111/ppl.70473)
Supplement: Supplementary file 2 — Table S1: List of excessive iron (Fe)‐responsive genes involved in different strategies as plant's defence system. [file PPL-177-e70473-s003.docx]

| Strategy | Gene Name | MSU_ID | RAP_ID | Regulation | Tissue | Function | Reference |
| --- | --- | --- | --- | --- | --- | --- | --- |
| I, II | OsIRT1  Table S1- List of excessive iron responsive genes involved in different tolerance strategies | LOC_Os03g46470 | Os03g0667500 | Down | Root | Act as a high-affinity iron transporter and plays a crucial role in the uptake of iron from the rhizosphere across the plasma membrane in the root. | Bashir *et al.* 2014, Stein *et al.* 2019, Kar *et al.* 2021, Regon *et al.* 2022 |
| I, II | OsYSL2 | LOC_Os02g43370 | Os02g0649900 | Down | Root  Shoot | Regulate iron transport to phloem and its translocation into the grain | Bashir *et al.* 2014, Aung *et al.* 2018, Wairich *et al.* 2021, Kar *et al.* 2021, Regon *et al.* 2022 |
| I, II | OsTOM1 | LOC_Os11g04020 | Os11g0134900 | Down | Root | Transporter of mugineic acid | Bashir *et al.* 2014, Stein *et al.* 2019, Kar *et al.* 2021, Regon *et al.* 2022 |
| I, II | OsYSL15 | LOC_Os02g43410 | Os02g650300 | Down | Root  Shoot | Ferric ion and DMA conjugate transporter | Bashir *et al.* 2014, Stein *et al.* 2019, Kar *et al.* 2021, Regon *et al.* 2022 |
| I, II | OsNRAMP1 | LOC_Os07g15460 | Os07g0258400 | Down | Root  Shoot | High affinity metal transporter | Quinet *et al.* 2012, Bashir *et al.* 2014, Aung *et al.* 2018, Stein *et al.* 2019, Wairich *et al.* 2021, Kar *et al.* 2021, Regon *et al.* 2022 |
| I | OsNAS1 | LOC_Os03g19427 | Os03g0307300 | Down | Root | Iron transporter | Inoue *et al.* 2003, Kar *et al.* 2021, Regon *et al.* 2022 |
| I | OsNAS2 | LOC_Os03g19420 | Os03g0307200 | Down | Root  Shoot | Iron transporter | Inoue *et al.* 2003, Quinet *et al.* 2012, Bashir *et al.* 2014, Aung *et al.* 2018, Stein *et al.* 2019, Wairich *et al.* 2021, Kar *et al.* 2021, Regon *et al.* 2022 |
| I, II | OsNAAT1 | LOC_Os02g20360 | Os02g0306401 | Down | Root  Shoot | Regulate biosynthesis of mugineic acid family phytosiderophores | Bashir *et al.* 2014, Finatto *et al.* 2015, Aung *et al.* 2018, Stein *et al.* 2019, Wairich *et al.* 2021, Kar *et al.* 2021, Regon *et al.* 2022 |
| I | OsDMAS1 | LOC_Os03g13390 | Os03g237100 | Down | Root | Regulate biosynthesis of mugineic acid family phytosiderophores | Bashir *et al.* 2014, Aung *et al.* 2018, Kar *et al.* 2021, Regon *et al.* 2022 |
| I | OsIRO2 | LOC_Os01g72370 | Os01g0952800 | Down | Root  Shoot | Regulate iron uptake from soil | Bashir *et al.* 2014, Aung *et al.* 2018, Wairich *et al.* 2021, Kar *et al.* 2021, Regon *et al.* 2022 |
| I | OsIMA1 | LOC_Os01g45914 | Os01g0647200 | Down | Root  Shoot | Regulate iron uptake and translocation | Regon *et al.* 2022 |
| I | OsHRZ1 | LOC_Os01g49470 | Os01g0689451 | -- | Root | Regulate iron acquisition | Peng *et al.* 2022 |
| I | OsHRZ2 | LOC_Os05g47780 | Os05g0551000 | -- | Root | Regulate iron acquisition | Peng *et al.* 2022 |
| I | OsFRO2 | LOC_Os04g48930 | Os04g0578600 | Down | Shoot | Ferric reductase oxidase | Bashir *et al.* 2014, Finatto *et al.* 2015, Aung *et al.* 2018, Wairich *et al.* 2021, Kar *et al.* 2021, Regon *et al.* 2022 |
| I | OsIRT2 | LOC_Os03g46454 | Os03g0667300 | Down | Root  Shoot | Fe²⁺ transporter | Bashir *et al.* 2014, Aung *et al.* 2018, Kar *et al.* 2021, Regon *et al.* 2022 |
| II, III | OsVIT2 | LOC_09g23300 | Os090396900 | Up | Root  Shoot | Vacuolar iron transporter | Finatto *et al.* 2015, Aung *et al.* 2018, Wairich *et al.* 2021, Kar *et al.* 2021 |
| II, III | OsVTL2 | LOC_Os04g45520 | Os04g0538400 | Up | Root  Shoot | Vacuolar iron transporter | Quinet *et al.* 2012, Bashir *et al.* 2014, Finatto *et al.* 2015, Wairich *et al.* 2021, Kar *et al.* 2021, Regon *et al.* 2022 |
| II, III | OsFER1 | LOC_Os11g01530 | Os11g0106700 | Up | Root  Shoot | Iron storage protein | Quinet *et al.* 2012, Stein *et al.* 2019, Wairich *et al.* 2021, Kar *et al.* 2021, Regon *et al.* 2022 |
| II, III | OsFER2 | LOC_Os12g01530 | Os12g0106000 | Up | Root  Shoot | Iron storage protein | Quinet *et al.* 2012, Stein *et al.* 2019, Wairich *et al.* 2021, Kar *et al.* 2021, Regon *et al.* 2022 |
| II, III | OsNAS3 | LOC_Os07g48980 | Os07g0689600 | Up | Root  Shoot | Nicotinamine synthase | Aung *et al.* 2018, Stein *et al.* 2019, Kar *et al.* 2021, Regon *et al.* 2022 |
| II | OsPEZ1 | LOC_Os03g37490 | Os03g0571900 | Up | Root | Efflux Phenolics | Regon *et al.* 2022 |
| II | OsRab6a | LOC_Os03g09140 | Os03g0191400 | Up | Shoot | Iron accumulation | Regon *et al.* 2022 |
| III | OsFRDL1 | LOC_Os03g11734 | Os03g0216700 | Up | Root | Citrate transporter | Aung *et al.* 2018 |
| III | OsFRDL2 | LOC_Os10g13940 | Os10g0206800 | Up | Root | Citrate transporter | Aung *et al.* 2018 |
| III | OsATM3 | LOC_Os06g03770 | Os06g0128300 | Up | Shoot | Mitochondrial ABC transporter essential for iron homeostasis | Regon *et al.* 2022 |
| III | OsMIT | LOC_Os03g18550 | Os03g0296800 | Up | Root  Shoot | Mitochondrial ABC transporter essential for iron homeostasis | Regon *et al.* 2022 |
| IV | OsGSNOR | LOC_Os02g57040 | Os02g0815500 | Up | Root | Root tolerance to iron toxicity | Aung and Masuda *et al.* 2018, Li *et al.* 2019 |
| IV | OsNAC4 | LOC_Os01g60020 | Os01g0816100 | Up | Shoot | Regulate severe iron toxicity tolerance | Finatto *et al.* 2015, 2018, Li *et al.* 2019 |
| IV | OsNAC5 | LOC_Os11g08210 | Os11g0184900 | Up | Shoot | Regulate severe iron toxicity tolerance | Finatto *et al.* 2015, 2018, Li *et al.* 2019 |
| IV | OsNAC6 | LOC_Os01g66120 | Os01g0884300 | Up | Shoot | Regulate severe iron toxicity tolerance | Finatto *et al.* 2015, 2018, Li *et al.* 2019 |
