# Supplementary material for: Meta‐Analysis of Iron Excess Stress in Rice: Genes and Mechanisms of Tolerance to Acidic Soil
Source: Physiol Plant. 2025 Aug 27;177(5):e70473. doi: 10.1111/ppl.70473 (PMC12391641; doi:10.1111/ppl.70473)
Supplement: Supplementary file 5 — Table S4: Summary of protein functions grouped in gene coexpression network. [file PPL-177-e70473-s002.docx]

Table S4- Summary of function of proteins grouped in coexpression network

| Coexpression Group | Gene | Function | Category | Reference |
| --- | --- | --- | --- | --- |
| I | DTX | Homologous DTX genes OsFRDL1 work as a citrate transporter required for efficient translocation of iron (Fe) | III | Yokosho *et al.* (2009) |
| I | LAC5 | As a member of laccase-like multicopper oxidases with ferroxidase activity, LAC5 might play a role in iron metabolism |  | Hoopes and Dean (2004) |
| II | VHA (A, B, C, D, E, F, G, H) – the peripheral V1 complex | Responsible for ATP hydrolysis to generate electrochemical gradient for secondary active transport, including Fe sequestration | III | Li *et al.,* (2022) |
| II | VHA (VHA-a, c, c′, c″, d, e)- membrane-integral V0 complex | Responsible for proton translocation to generate electrochemical gradient for secondary active transport, including Fe sequestration | III | Li *et al.,* (2022) |
| II | ATPC1 | Regulate ATPase activity and the flow of protons across membrane of chloroplast |  | Dal Bosco *et al.* (2004) |
| II | ADH2/GSNOR1 | A major bioactive source of nitric oxide (NO) | IV | Wang *et al.* (2022) |
| II | ATP5 (OSCP subunit) | Mitochondrial ATP synthase subunits |  | Robison *et al.* (2009) |
| II | PDE334 | Involved in ATP synthesis |  | Berardini *et al.* (2015) |
| III | ABCG | Play a crucial role in substance transportation | I | Dhara and Raichaudhuri, (2021) |
| III | FREE1 | A plant-specific component of the endosomal sorting complex required for transport (ESCRT), important for multivesicular body biogenesis. It decreases Fe absorption in plants under Fe-deficient growth conditions by apolar localization of IRT1 on plasma membrane | I | Barberon *et al.* (2014) |
| III | ISU1 | Regulates iron homeostasis in the mitochondria |  | Tone *et al.* (2004) |
| III | GRXS17 | Maintain redox homeostasis. |  | Cheng *et al.* (2020) |

*Member of coexpression group IV discussed in Table S1.
