# Supplementary material for: Meta‐Analysis of Iron Excess Stress in Rice: Genes and Mechanisms of Tolerance to Acidic Soil
Source: Physiol Plant. 2025 Aug 27;177(5):e70473. doi: 10.1111/ppl.70473 (PMC12391641; doi:10.1111/ppl.70473)
Supplement: Supplementary file 6 — Table S5: Subcellular localization, signal, and membrane types of Iron (Fe) excess‐responsive genes. [file PPL-177-e70473-s006.docx]

Table S5- Subcellular localization, signal, and membrane types of iron excess-responsive genes

| Protein_ID | Symbol (Strategy) | Localizations | Signals | Membrane types |
| --- | --- | --- | --- | --- |
| Os03g0667500 | OsIRT1  (I, II) | Cell membrane\|Lysosome/Vacuole | Signal peptide\|Transmembrane domain | Transmembrane |
| Os02g0649900 | OsYSL2  (I, II) | Cell membrane | Signal peptide\|Transmembrane domain | Transmembrane |
| Os11g0134900 | OsTOM1  (I, II) | Cell membrane | Transmembrane domain | Transmembrane |
| Os02g0650300 | OsYSL15  (I, II) | Cell membrane | Transmembrane domain | Transmembrane |
| Os07g0258400 | OsNRAMP1  (I, II) | Cell membrane\|Lysosome/Vacuole | Transmembrane domain | Transmembrane |
| Os03g0307300 | OsNAS1  (I) | Cytoplasm | -- | Soluble |
| Os03g0307200 | OsNAS2  (I) | Cytoplasm | -- | Soluble |
| Os02g0306401 | OsNAAT1  (I) | Cytoplasm | -- | Soluble |
| Os03g0237100 | OsDMAS1  (I) | Cytoplasm | Nuclear export signal | Soluble |
| Os01g0952800 | OsIRO2  (I) | Nucleus | Nuclear localization signal | Soluble |
| Os01g0647200 | OsIMA1  (I) | Cytoplasm\|Nucleus | -- | Soluble |
| Os01g0689451 | OsHRZ1  (I) | Cytoplasm | Nuclear export signal | Soluble |
| Os05g0551000 | OsHRZ2  (I) | Cytoplasm | Nuclear export signal | Soluble |
| Os04g0578600 | OsFRO2  (I) | Cell membrane | Signal peptide\|Transmembrane domain | Transmembrane |
| Os03g0667300 | OsIRT2  (I) | Cell membrane\|Lysosome/Vacuole | Signal peptide\|Transmembrane domain | Transmembrane |
| Os09g0396900 | OsVIT2  (II, III) | Lysosome/Vacuole | Transmembrane domain | Transmembrane |
| Os04g0538400 | OsVTL2  (II, III) | Cell membrane | Transmembrane domain | Transmembrane |
| Os11g0106700 | OsFER1  (II, III) | Plastid | Chloroplast transit peptide | Soluble |
| Os12g0106000 | OsFER2  (II, III) | Plastid | Chloroplast transit peptide | Soluble |
| Os07g0689600 | OsNAS3  (II, III)) | Cytoplasm | -- | Soluble |
| Os03g0191400 | OsRab6a  (II) | Lysosome/Vacuole\|Golgi apparatus | -- | Lipid anchor |
| Os03g0571900 | OsPEZ1  (III) | Cell membrane\|Lysosome/Vacuole | Transmembrane domain | Transmembrane |
| Os03g0216700 | OsFRDL1  (III) | Cell membrane | Transmembrane domain | Transmembrane |
| Os10g0206800 | OsFRDL2  (III) | Cell membrane | Transmembrane domain | Transmembrane |
| Os06g0128300 | OsATM3  (III) | Mitochondrion | Mitochondrial transit peptide | Transmembrane |
| Os03g0296800 | OsMIT  (III) | Mitochondrion | -- | Transmembrane |
| Os02g0815500 | OsGSNOR  (IV) | Cytoplasm | -- | Soluble |
| Os01g0816100 | OsNAC4  (IV) | Nucleus | Nuclear localization signal\|Nuclear export signal | Soluble |
| Os11g0184900 | OsNAC5  (IV) | Nucleus | Nuclear localization signal | Soluble |
| Os01g0884300 | OsNAC6  (IV) | Nucleus | Nuclear localization signal\|Nuclear export signal | Soluble |
